# Preparation and Transport Performances of High-Density, Aligned Carbon Nanotube Membranes

**DOI:** 10.1186/s11671-015-0970-8

**Published:** 2015-06-19

**Authors:** Lei Zhang, Bin Zhao, Chuan Jiang, Junhe Yang, Guangping Zheng

**Affiliations:** School of Materials Science and Engineering, University of Shanghai for Science and Technology, Shanghai, 200093 China; Department of Mechanical Engineering and Shenzhen Research Institute, The Hong Kong Polytechnic University, Hung Hom, Kowloon, Hong Kong

**Keywords:** Carbon nanotubes, Nano-filtration membrane, Gas transport, Liquid transport, Separation

## Abstract

We report a simple and effective method for the preparation of high-density and aligned carbon nanotube (CNT) membranes. The CNT arrays were prepared by water-assisted chemical vapor deposition (CVD) and were subsequently pushed over and stacked into dense membranes by mechanical rolling. It was demonstrated that various gases and liquids, including H_2_, He, N_2_, O_2_, Ar, water, ethanol, hexane, and kerosene, could effectively pass through the aligned carbon nanotube membranes. The membranes exhibited different selections on different gases, indicating that there was a separation potential for the gas mixtures. The selectivities (H_2_ relative to other gases) of H_2_/He, H_2_/N_2_, H_2_/O_2_, and H_2_/Ar were found to be lower than that of the ideal Knudsen model. For pure water, the permeability was measured to be 3.23 ± 0.05 ml·min^−1^·cm^−2^ at 1 atm, indicating that the CNT membranes were promising for applications in liquid filtration and separation.

## Background

In the past decade, vertically aligned carbon nanotube (VACNT) membranes where carbon nanotubes are sealed in a polymer or inorganic matrix have been developed for gas and liquid transport applications [1–7]. It is well known that those composite membranes suffer from a trade-off between selectivity and permeability. In some cases, they are even susceptible to fouling or exhibit low chemical resistance. Hinds et al. [1] first fabricated multi-walled VACNT membranes with an inner diameter of 6–7 nm embedded in a rigid polystyrene matrix. They demonstrated that liquid transporting through the composite membrane was several orders of magnitude faster than that predicted by the classical hydrodynamics theory owing to the smooth CNT walls. Holt et al. [2] adopted a micro-fabrication method to produce membranes in which the double-walled CNTs were used as the only pores to span through a silicon nitride matrix deposited by chemical vapor deposition (CVD). They found that the measured gas flow was more than one order of magnitude larger than that predicted from the Knudsen diffusion model. In spite of their smaller pore sizes, the gas and water permeabilities of those nanotube-based membranes were several orders of magnitude greater than those of the commercial polycarbonate membranes. Kim et al. [3] reported the results of gas mixture transporting through the single-walled CNT membrane with an average pore size of 1.2 nm. The aligned single-walled CNTs were filtrated with a poly(tetrafluoroethylene) filter and the spaces among the CNTs were then sealed with polysulfone polymer by spin coating. They confirmed that non-Knudsen transport could occur in the aligned CNT membranes and found that the permeabilities of CO_2_ and CH_4_ passing through the membrane with additional polymer coating were lower than those predicted from the Knudsen diffusion model. The reduction in permeability was found to be proportional to the transport resistance offered by the additional polymer layer.

The previously reported composite membranes had low CNT porosity since their fractions of CNT permeation areas were only 0.079–2.7 %. Although the fluxes passing through the individual nanotubes were high, the fluxes of membrane areas were limited because of the low porosity. In addition, the fabrication processes of the composite CNT membranes were expensive and complicated. In order to achieve more efficient and cost-effective purification, advanced membrane technologies with controlled and novel pore architectures have to be developed. In contrast to the above studies, which used CNT pores as transport pathways, Srivastava et al. [6] made a CNT filter from high-density and vertically aligned CNT forests without a filler. Because their nanotubes were mostly blocked by catalyst particles, transport was in the interstitial spaces, which were approximately 20–30 nm across. Yu et al. [7] fabricated a freestanding VACNT membrane with high packing density by shrinking VACNT arrays, and found that gas permeances based on total membrane area were 1–4 orders of magnitude higher than VACNT membranes in the literature, which highlights the potential of high-density CNT membranes in mass transport.

In this work, a facile and effective method is developed to prepare high-density, aligned, and freestanding CNT membranes by mechanically rolling and densifying VACNT arrays. The membrane structure is characterized and transport performances of some gases and liquids across the membrane are investigated. Compared with the buckypaper membrane [8], this large area and aligned CNT membrane, which employ the narrow spacing among aligned CNTs as mass transport pathway, have more ordered pore structure.

## Methods

Growth of VACNTs was conducted by a water-assisted CVD technique by using Fe(1.4 nm)/Al_2_O_3_(40 nm)/Si as the catalyst [9, 10]. High-purity ethylene (99.99 %) was used as carbon source and Ar/H_2_ (99.999 %) were used as carrier gases with a total flow rate of 650 sccm. During the growth process, a controlled amount of water vapor was employed as catalyst preserver and enhancer and was supplied by passing a portion of the carrier gas Ar through a water bubbler [11–14]. Typically, VACNT array was grown at 815 °C with ethylene (100 sccm) under a water concentration of 100–200 ppm for 10 min [15].

Two glass slides were used as the simple tool to fabricate CNT membrane. Firstly, the as-grown CNT array was fixed by gluing the Si substrate on a glass slide. Secondly, another slide was put on top of the CNT array and used as a guide for the subsequent shear pressing from a roller. During the shear pressing, CNTs were forced down to one direction. Then, the aligned CNT membranes were peeled from the substrate by ultrasonication in deionized water. After drying in vacuum at 60 °C for 4 h, freestanding and aligned CNT membranes were obtained.

The freestanding CNT membrane was first sealed between two pieces of aluminum adhesive tapes with pre-punched holes (3 mm in diameter) [10]. Then the membrane was mounted in the gas line of a permeation testing apparatus, which was purged with the target gas for several times to avoid any possible impurities [16–18]. Finally, pure H_2_, He, N_2_, Ar, O_2_, or CO_2_ (99.999 %) were introduced to the upstream side of the membrane [19–22] for permeation measurements. A pressure or flow controller (MKS 250E) was connected to the upstream and downstream sides of the composite membrane to control the relative gas pressures by automatically tuning the gas feeding rates. The permeabilities at a variety of pressures (10–100 Torr) were measured using a mass-flow meter connected at the downstream side.

The transport properties of liquid (water, ethanol, hexane, and kerosene) were measured in a liquid-collecting device, and a permeate sample was weighed every 1 h to determine the flux [23–25]. The pressures were 10–100 Torr on the permeate side. All the measurements were carried out at room temperature.

## Results and Discussion

**Fig. 1:**
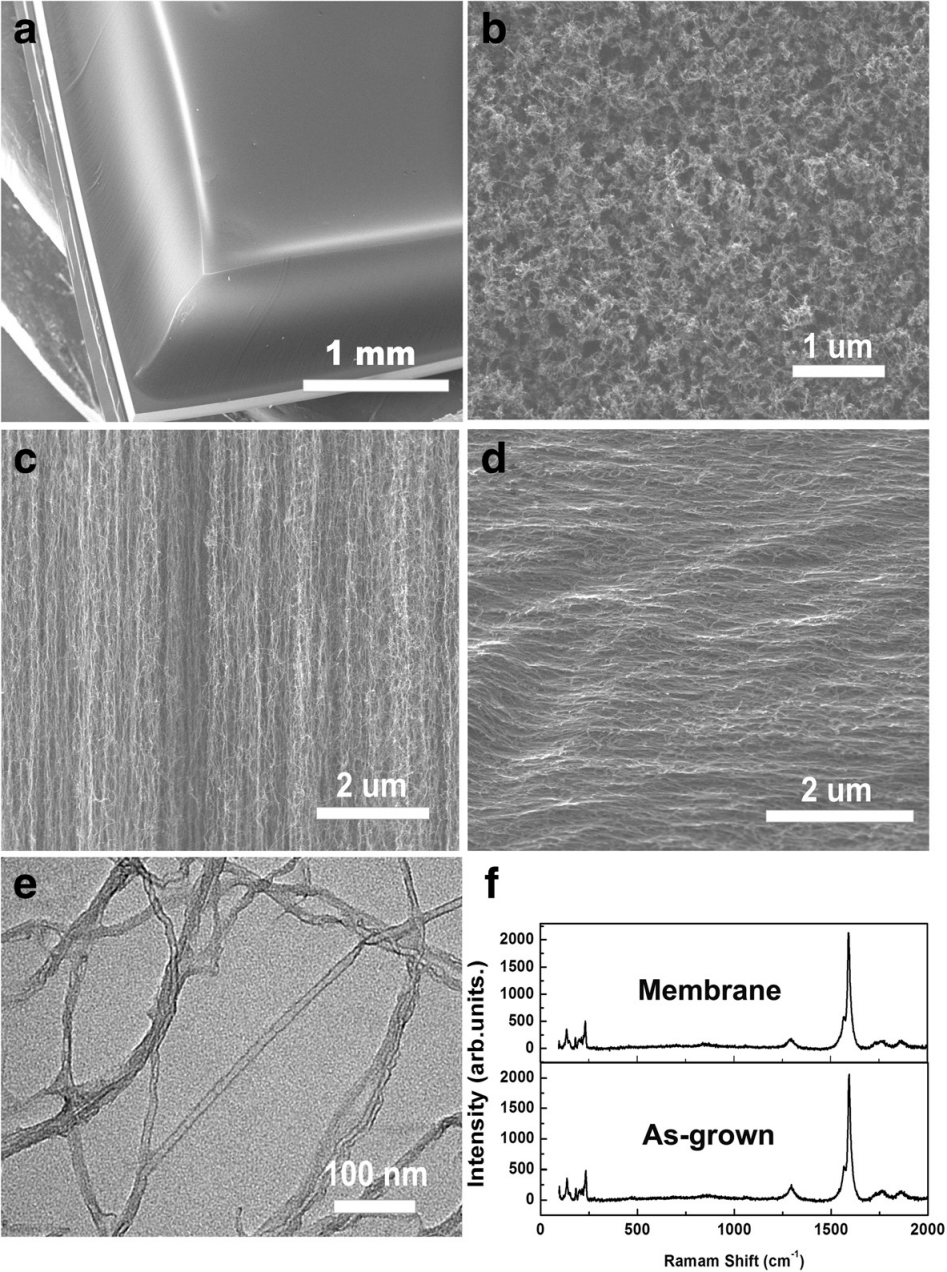
Characterization of the CNT arrays. **a** Cross-sectional SEM image of the VACNT array with 1 mm in thickness. **b** SEM image of the VACNT array surface under high-resolution magnification. **c**, **d** Cross-sectional SEM images of VACNT arrays before and after rolling down, respectively. **e** A representative TEM image for the determination of the CNT diameter, showing an inner diameter of about 5 nm. **f** Raman spectra of the VACNT membrane before and after rolling down

Figure 1a shows side-view SEM image of a typical VACNT array grown by water-assisted CVD, which is about 1-mm tall. As shown in Fig. 1b, the as-grown sample presents a typical morphology of vertically aligned CNT forests with spaghetti-like surface, and no cracks are observed. Side-view SEM observation shows the alignment of the as-grown CNT array (Fig. 1c). Close examination reveals that the CNTs are entangled with each other inspite of the overall alignments. After the CNT layer was rolling down by a piece of glass slide, the CNTs were still aligned but were packed into a more dense structure (Fig. 1d). Figure 1e shows the high-resolution transmission electron microscopy (HRTEM) image of a typical CNT with a diameter of about 5 nm. It can be found that the number of its graphitic walls is about 3. TEM examination could demonstrate the high purity of the nanotubes since no metal catalysts are observed. Raman spectroscopy was employed to characterize the structure of CNTs in the membrane at different stages of fabrication. Figure 1f shows the Raman spectra of the as-synthesized CNT array and the CNT membrane. A G-band at 1592 cm^−1^ and a D-band at 1295 cm^−1^ are found in Raman spectra of the samples, which are caused by the in-plane vibration of graphite with an E_2g_-symmetry intra-layer mode and the defects in the nanotubes or amorphous carbon, respectively. The I_G_/I_D_ ratio of 12.3 for the as-synthesized CNTs suggests a good crystallinity of the CNT array grown by water-assisted CVD. After mechanical rolling, the I_G_/I_D_ ratio shows negligible change, indicating that the structure of CNTs has not been destroyed during the fabrication of the CNT membrane.

**Fig. 2:**
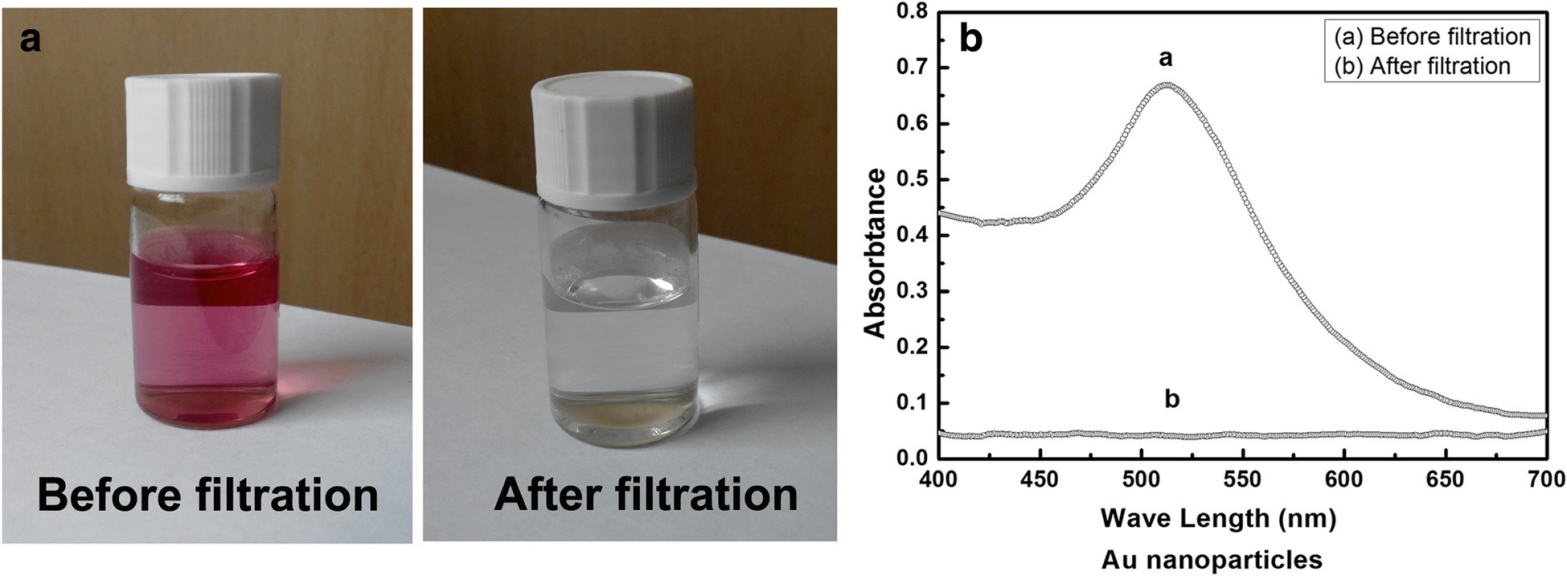
**a**, **b** Optical images and UV–vis spectra of the nano-gold solution on the feed and permeate sides of a CNT membrane after the size exclusion experiment

Size exclusion experiments were performed by filtering 10 nm Au nanoparticles in DI water. Red-colored water solution with 10 ppm Au was introduced to the feed side of the dense CNT membrane, which was pressurized to 50 torr. The permeated liquid showed no color, indicating that the Au nanoparticles were completely blocked by the CNT membrane, as shown in Fig. 2a. Figure 2b shows UV–vis spectra of the feed Au solution and the permeated liquid. The characteristic surface plasmon resonance band around 510 nm of the Au nanoparticles, seen for the feed solution, disappeared completely for the permeated solution collected after filtration, suggesting the complete block of the Au nanoparticles of 10 nm. All these results provide direct and important evidence that the pores of the high-density and aligned CNT membrane were indeed smaller than 10 nm. Compared with the intertube distance of as-grown CNT array [11, 13], the spacing among nanotubes in the CNT membrane shrank at least two times, indicating the high-density of the CNT membrane made by crashing VACNTs.

**Fig. 3:**
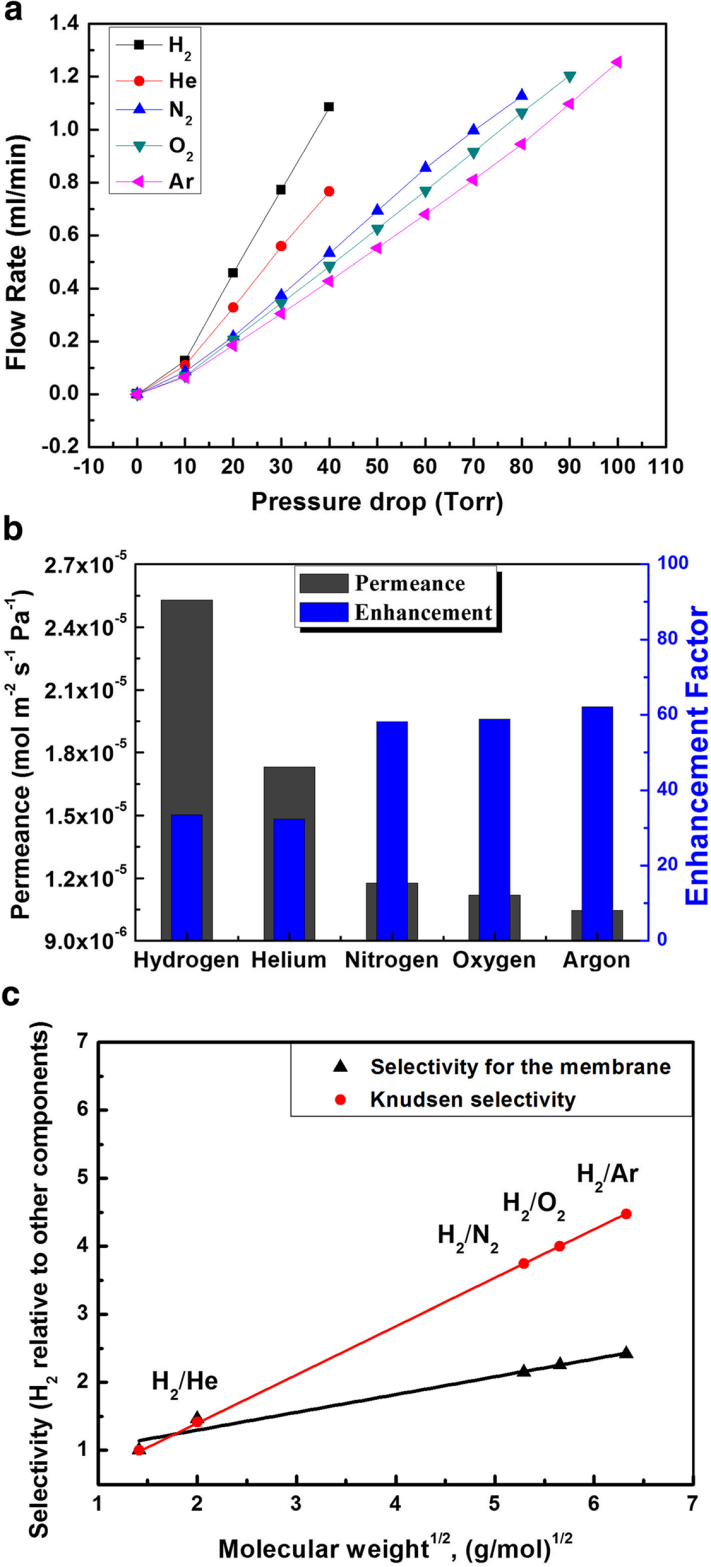
Gas permeation performance of VACNT membranes. **a** The flow rates of gases passing through the CNT membrane are scaled linearly with the pressure drop. **b** Histogram of the observed permeance and enhancement factors over Knudsen diffusion for each gas. **c** Selectivity of H_2_ relative to other gases of the CNT membranes

As shown in Fig. 3a, the flow rate increases with feed pressure slightly, which means there is contribution of viscous flow to total gas transport. Although the average pore size is less than 10 nm, the CNT membranes may contain a few large pores for viscous flow. Thus, both viscous and Knudsen flow influence the transport across these membranes. KCl diffusion experiments [10, 21] were performed to estimate the membrane porosity which was determined to be ~0.0428. The enhancement factor was defined as the ratio of experimental permeance to the calculated Knudsen permeance. Figure 3b shows the experimental permeance for H_2_, He, N_2_, O_2_, and Ar gases passing through the aligned CNT membrane, and the enhancement factor over Knudsen diffusion. It was worth emphasizing that the experimentally measured gas permeability is scaled with respect to the molecular weight of the gas with an exponent of 0.3, which is lower than that (0.5) predicted by the Knudsen diffusion model. The deviation from the ideal Knudsen model in CNT membrane may be caused by the smooth surface properties of the CNTs, which may play an important role in the transport of gas molecules through the aligned CNT membranes [26].

In general, gas transport through a porous material can be described by a combination of viscous flow, Knudsen diffusion, and surface diffusion [10, 16, 21, 24]. In gas separations, higher flux with lower separation for viscous flow and lower flux with higher separation for surface diffusion can be obtained. However, the permeance will increase with the pressure drop when viscous flow occurs [16]. For most gases at room temperature, their mean-free paths are significantly larger than the pore size of our CNT membranes. Knudsen diffusion becomes prominent when the mean-free path of the diffusing species is larger than the pore diameter. Hence, one would expect that the gas transporting through the CNT membrane could be consistent with the Knudsen diffusion whose permeance is estimated as

1 $$ {P}_{\mathrm{Kn}}=\frac{\varPhi {\varepsilon}_{\mathrm{p}}}{\tau RTL}{\left(\frac{8RT}{\pi M}\right)}^{0.5}, $$

where *P*_Kn_ is the Knudsen permeation (mol m^−2^ s^−1^ Pa^−1^), *ε*_p_ is the porosity, *τ* is the tortuosity, *Φ* is the inner diameter of CNT (m), *L* is the layer thickness (m), *M* is the molecular mass (kg mol^−1^) of the gas molecule, and *T* is the absolute temperature (K).

The permeances of the five gases range between 1.0 × 10^−5^ and 2.5 × 10^−5^ mol m^−2^ s^−1^ Pa^−1^, and enhancement factors ranged between 30 and 60, indicating a much higher transport rate than Knudsen diffusion. The selectivities (H_2_ relative to other gases) were lower than Knudsen for dense CNT membranes, as shown in Fig. 3c. These results clearly indicate that the high permeances through these dense CNT membranes are not Knudsen [17]. Surface diffusion and viscous flow may play important roles in the transport of gas molecules through the aligned CNT membranes. From the perspective of separations, lower flux with higher separation for surface diffusion and higher flux with lower separation is obtained for viscous flow. The low gas selectivities indicate the presence of viscous flow in the membrane. Although the average pore diameter is less than 10 nm, the CNT membranes may have a few large pores that favor high molecule weight gases permeation. Furthermore, the smooth surface of the CNTs may also play a role in the selectivity derivation of gas molecules from the Knudsen diffusion.

Pressure-driven liquid transport through the CNT membranes was measured in a pressure flow membrane transport device [10]. Briefly, the membrane was assembled in the flow cell, and the nitrogen provided the required pressure to drive the liquid through the membrane. Transport behavior of water, ethanol, hexane, and kerosene were measured, and all the measurements were carried out at a constant temperature. For measurements of different liquids on the same membrane, the sample is dried in air for 12 h. The weight of the liquid permeating through the membrane was measured at intervals of 1 h over a period of 12 h. And then the volume of permeated liquid was determined based on the weight difference.

Liquid flows passing through the porous membranes can be predicted using the Hagen-Poiseuille equation given by

2 $$ {\mathrm{Q}}_{\mathrm{HP}}=\frac{\pi {\left(d/2\right)}^4}{8\mu}\frac{\Delta p}{L}, $$

where *Q*_HP_ is the volumetric flow rate (ml min^−1^ cm^−2^ atm^−1^), Δ*p* is the pressure drop (Torr), *d* is the membrane diameter (nm), *μ* is the dynamic viscosity, and *L* is the membrane thickness (μm).

The implication of Equation 2 for nanoporous materials is that the transport rate is limited by the pressure drop across the pores. The parameters of the high-density and aligned CNT membranes are listed in Table 1 for the calculation of the permeance of liquid. The enhancement factor is defined as the ratio of the measured permeance to the calculated Hagen-Poiseuille flow rate.

**Table 1:** Parameters of the high-density and aligned CNT membranes

Membrane pore size (nm)	*Δp* (Torr)	Thickness *L* (μm)	Dynamic viscosity *μ* (Pa·s)	CNT areal density (cm^−2^)	Membrane area (cm^2^)
10	100	~120	0.307~1.675 × 10^−3^	~10^11^	7.065 × 10^−2^

**Fig. 4:**
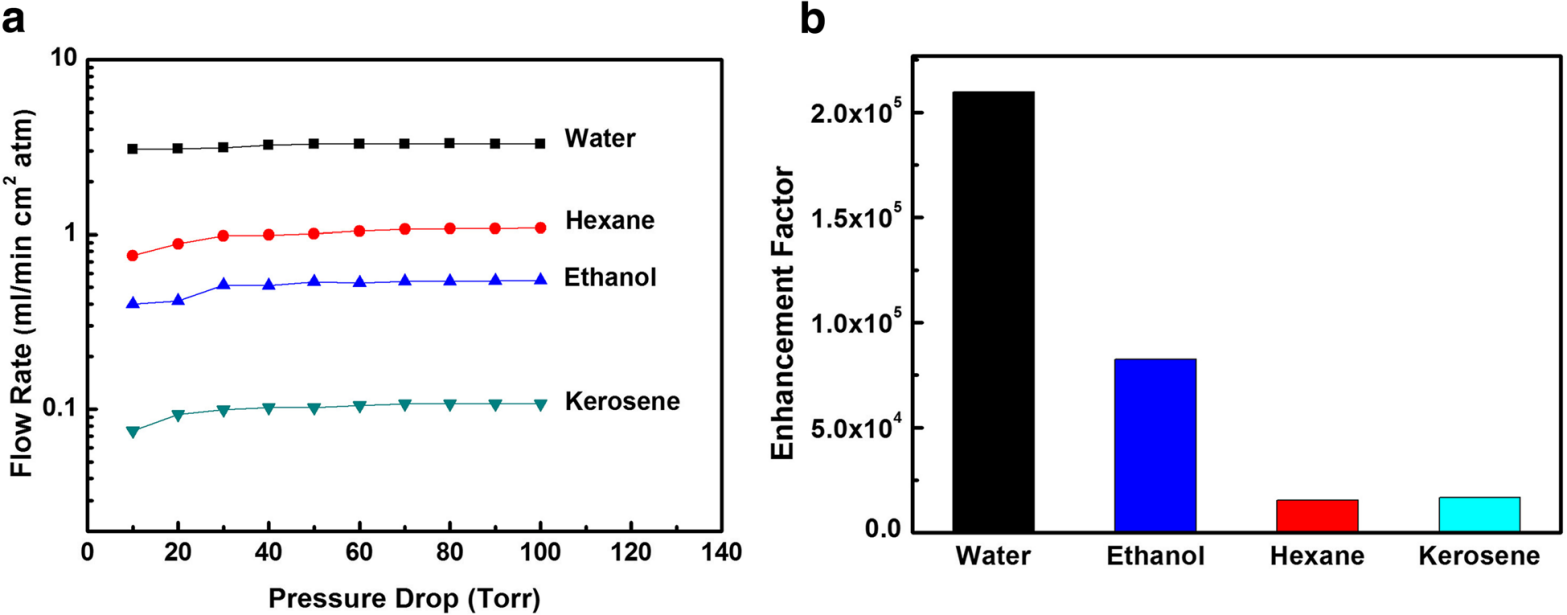
Liquid permeation performance of VACNT membranes. **a** The experimental permeance of each liquid, and **b** the enhancement factor of the CNT membranes

Figure 4a shows flow rates of water, hexane, ethanol, and kerosene transporting through the high-density and aligned CNT membrane under different pressure drops. The measured liquid flow rates reveal that they are more than 4–5 orders of magnitude faster than the hydrodynamic flow rate calculated from the Hagen-Poiseuille equation (Fig. 4b). Such trend of change in flow velocities could not be explained by conventional parameters such as the viscosity and hydrophobicity. For instance, water passes through the membrane very quickly, while hexane, ethanol, and kerosene pass slowly. Although hexane is less viscous and more hydrophobic to the channels than water, the hexane flow rate through the dense CNT membrane is lower than that of water. The possible reason is that water shows a different structure from other solvents, the “free” OH bond of water combines with the nanotube wall to form a depletion layer [2], which reduces the number of hydrogen bonds in the depletion layer to reduce the friction and enhance the flow rate. On the other hand, even though ethanol may also form a depletion layer with the nanotube wall, each ethanol molecule has only one OH bond, which induces a relatively weak hydrogen bond effect with respect to water [25]. Besides, the molecular diameter of a single ethanol molecule is much larger than that of a water molecule, the clustering may significantly increase the viscosity, thus the flow rate of ethanol is lower than that of water. As for kerosene, the viscous and the molecular diameter are much larger than those of water molecule, the flow rate through the dense CNT membrane is lower than that of water.

Table 2 compares solvent permeability of our high-density and aligned CNT membrane with the VACNT composite membranes in literatures. For all the liquids measured here, our permeability results are higher than those of VACNT/polystyrene [21, 27], polycarbonate, and CNT/Si_3_N_4_ membranes [2]. Our high-density CNT membrane shows similar water permeability, higher ethanol permeability but lower hexane permeability with respect to the CNT/epoxy membrane [25]. The structure and the pore size distribution of the membranes may lead to the different permeabilities. For the VACNT composite membranes, the space between CNTs were sealed by polymeric or ceramic materials, and only the opened CNTs act as the channels for liquid transport. So the available pores for permeation are limited, and the overall flow rate will be low. Our dense CNT membrane employs all the space among CNTs as permeation pores of liquid (higher porosity), thus the overall permeance is much higher. It is also worth noting that our dense and aligned CNT membrane exhibits high water/kerosene selectivity, indicating potential application of the CNT membrane in water/oil separation.

**Table 2:** Permeability of different liquids passing through the CNT membranes^a^

	This research	CNT/epoxy [25]	CNT/polystyrene [21]	MWNT/polystyrene [27]	Polycarbonate membrane [2]	CNT/Si_3_N_4_ [2]
Pore size (nm)	10	10	7	7	15	1.3–2.0
Length (μm)	120	4000	34~126	3~70	6	2~3
Water permeability	3.23	3.9	0.78	0.59~1.02	~6 × 10^−3^	(1.2~4.6) × 10^−1^
Ethanol permeability	0.507	6.3 × 10^−4^	0.35	0.35		
Hexane permeability	1.00	9.3	0.45	0.44		
Kerosene permeability	0.10					

^a^Permeability at 1 atm is in the units of ml min^−1^ cm^−2^

## Conclusions

In summary, we have demonstrated a simple and effective method to prepare high-density and aligned CNT membranes, which have advantages over other CNT composite membranes. The average spacing between CNT membranes was ~10 nm after rolling. Remarkably, the mechanical rolling did not destroy the aligned structure of CNTs or introduce other defects, and the membranes’ aligned structure remained unchanged. The CNT membranes show significantly high flow rates for the transports of various gases and liquids including H_2_, He, N_2_, O_2_, Ar, water, ethanol, hexane, and kerosene. The gas permeability of the high-density and aligned CNT membrane is much higher than the Knudsen permeability and is scaled with respect to the molecular weight of the gases with an exponent lower than that predicted by the Knudsen diffusion model. Moreover, it was found that different samples with the same preparation conditions kept a good consistency in the permeances of gases that the flow rate increased with increasing pressure drop. This phenomenon of deviation confirms the existence of a non-Knudsen transport and a thermally activated diffusion process. The membranes exhibited different selections on different gases, indicating that there was a separation potential for the gas mixtures. The selectivities of H_2_/He, H_2_/N_2_, H_2_/O_2_, and H_2_/Ar were found to be lower than that of the ideal Knudsen model. For pure water, the permeability was measured to be 3.23 ± 0.05 ml·min^−1^·cm^−2^ at 1 atm, indicating that the CNT membranes were promising for applications in liquid filtration and separation. In addition, the CNT membranes are found to have excellent filtration performance in nano-gold solution.
